# Analyses of Extended-Spectrum-β-Lactamase, Metallo-β-Lactamase, and AmpC-β-Lactamase Producing Enterobacteriaceae from the Dairy Value Chain in India

**DOI:** 10.3390/antibiotics12091449

**Published:** 2023-09-14

**Authors:** Tushar Kumar Dey, Johanna Frida Lindahl, Åke Lundkvist, Delia Grace, Ram Pratim Deka, Rajeswari Shome, Samiran Bandyopadhyay, Naresh Kumar Goyal, Garima Sharma, Bibek Ranjan Shome

**Affiliations:** 1 Department of Biosciences, International Livestock Research Institute, Nairobi 00100, Kenya; 2 Zoonosis Science Center, Department of Medical Biochemistry and Microbiology, Uppsala University, 75123 Uppsala, Sweden; 3 ICAR-National Institute of Veterinary Epidemiology and Disease Informatics, Bengaluru 560064, India; 4 Department of Clinical Sciences, Swedish University of Agricultural Sciences, 75007 Uppsala, Sweden; 5 Food and Markets Department, Natural Resources Institute, Chatham Maritime ME4 4TB, UK; 6 International Livestock Research Institute, Regional Office for South Asia, New Delhi 110012, India; 7 Eastern Regional Station, ICAR-Indian Veterinary Research Institute, Kolkata 700037, India; 8 Dairy Microbiology Division, National Dairy Research Institute, Karnal 132001, India

**Keywords:** antimicrobial resistance, AMR, enterobacteriaceae, β-lactamase, ESBL, MBL, AmpC, dairy milk, intensification, food safety

## Abstract

The consumption of milk contaminated with antibiotic-resistant bacteria poses a significant health threat to humans. This study aimed to investigate the prevalence of Enterobacteriaceae producing β-lactamases (ESBL, MBL, and AmpC) in cow and buffalo milk samples from two Indian states, Haryana and Assam. A total of 401 milk samples were collected from dairy farmers and vendors in the specified districts. Microbiological assays, antibiotic susceptibility testing, and PCR-based genotyping were employed to analyze 421 Gram-negative bacterial isolates. The overall prevalence of β-lactamase genes was 10% (confidence interval (CI) (7–13)), with higher rates in Haryana (13%, CI (9–19)) compared to Assam (7%, CI (4–11)). The identified β-lactamase genes in isolates were bla_CMY_, bla_MOX_, bla_FOX_, bla_EBC_, and bla_DHA_, associated with AmpC production. Additionally, bla_CTX-M1_, bla_SHV_, and bla_TEM_ were detected as ESBL producers, while bla_VIM_, bla_IMP_, bla_SPM_, bla_SIM_, and bla_GIM_ were identified as MBL producers. Notably, *Shigella* spp. were the dominant β-lactamase producers among identified Enterobacteriaceae. This study highlights the presence of various prevalent β-lactamase genes in milk isolates, indicating the potential risk of antimicrobial-resistant bacteria in dairy products. The presence of β-lactam resistance raises concern as this could restrict antibiotic options for treatment. The discordance between genotypic and phenotypic methods emphasizes the necessity for comprehensive approaches that integrate both techniques to accurately assess antibiotic resistance. Urgent collaborative action incorporating rational and regulated use of antibiotics across the dairy value chain is required to address the global challenge of β-lactam resistance.

India is the world’s largest milk-producing country, with 24% of the world’s milk output [1], leading over the USA, China, Pakistan, and Brazil [2]. Milk serves as a vital source of animal protein, particularly in a country such as India, where a substantial population follows a vegetarian diet. Additionally, it plays a crucial role in generating income and employment opportunities for countless households engaged in the milk value chain [2].

## 1. Introduction

In India, the majority (around 80%) of milk is produced by smallholder dairy farmers and traded through an unorganized dairy sector [3]. While this sector provides economic and social advantages, it also poses food safety concerns [4]. Unorganized dairy farms often lack essential resources, infrastructure, and access to animal health services [5,6,7]. Milk plays a significant role in the transmission of various foodborne bacteria from animals to humans [8]. Consuming milk contaminated with antimicrobial residues [9] or resistant bacteria poses a potential hazard for the spread of antimicrobial resistance (AMR).

Antibiotics play a crucial role in India’s dairy sector, serving as a means of disease control and ensuring the overall welfare of animals to sustain productivity. Moreover, they are frequently employed prophylactically or metaphylactically in India and other low and middle-income countries (LMIC) to prevent infections and promote growth and production [10,11,12]. While antibiotics can enhance the health and productivity of dairy animals, their usage can also foster the emergence of resistant strains [13]. The irrational and non-therapeutic use of antibiotics in farm animals can have a substantial impact on public health [14,15].

In livestock, β-lactam antibiotics are widely used for treating bacterial infections [16]. However, bacterial β-lactamases possess the capacity to hydrolyze the β-lactam ring found in these antibiotics. Although third-generation cephalosporins and aztreonam exhibit relative resistance to most bacterial β-lactamases, the emergence of extended-spectrum-β-lactamase (ESBL) poses a challenge. ESBL-producing bacteria have acquired the capability to hydrolyze these antibiotics as well [17,18].

ESBL-producing Gram-negative bacteria (GNB) pose a significant challenge as they often demonstrate resistance to multiple drug classes, including aminoglycosides, cotrimoxazole, tetracycline, and fluoroquinolones [18]. This multidrug resistance pattern adds complexity to treatment options and underscores the critical need to curb the further spread of ESBL-producing bacteria to preserve the efficacy of multiple antibiotics against these pathogens.

Another concern pertains to GNB that produces AmpC-β-lactamases, capable of inactivating a broad spectrum β-lactam antibiotics, including penicillins, cephalosporins, and monobactams [19]. Carbapenems, on the other hand, are broad-spectrum antibiotics used to combat GNB infections resistant to penicillins or cephalosporins [20], and severe bacterial infections caused by β-lactamase-producing GNB [21]. However, resistance to carbapenems has emerged due to their extensive and indiscriminate use, inadequate sanitation practices, and population growth [22].

The rapid global spread of GNB carrying plasmid-mediated ESBL, AmpC and MBL poses a significant clinical concern [23,24,25]. The presence of GNB with β-lactam resistance in milk, holds significant importance in the Indian subcontinent. This is due to the fact that some individuals consume raw milk for its perceived health benefits, and a considerable number of people regularly consume naturally fermented milk as lassi [26].

Food-producing animals, particularly cattle, have been identified as the primary source of antibiotic-resistant pathogenic bacteria within the Enterobacteriaceae family across multiple countries [27,28,29,30,31,32,33]. Among the most encountered members of Enterobacteriaceae in this category is *Escherichia coli, Klebsiella* spp., *Shigella* spp., and *Salmonella* spp., owing to their role in causing diseases in dairy cattle [34,35,36]. Investigating β-lactam resistance in these bacteria is of utmost importance to address and manage the emergence and spread of antibiotic-resistant strains among food-producing animals, a critical measure for safeguarding both animal and human health.

Previous studies on antibiotic resistance has predominantly focused on human populations, with limited attention given to animals [13,37]. Even when studies involving animals were conducted, they predominantly focused on clinical scenarios involving dairy animals in India and other countries [38,39,40,41,42,43,44]. Consequently, there exists a dearth of evidence concerning bacteria carrying resistance genes in milk intended for human consumption. Thus, the objective of this study is to assess the prevalence of β-lactamases in the dairy value chain. Our study aimed to evaluate antibiotic resistance in milk obtained from farms and points of sale, intended for human consumption to address the gap in knowledge of the exposure to consumers.

To achieve this, we conducted a cross-sectional study in two Indian states, Haryana and Assam, with the objective of investigating the occurrence of ESBLs, AmpC, and MBLs in Enterobacteriaceae isolated from dairy milk meant for consumption. To achieve this, both pasteurized milk and raw milk samples were included in the analysis. Furthermore, our study encompassed an assessment of the concordance and discordance between phenotypic and genotypic methods employed to identify β-lactam resistance within milk isolates.

## 2. Results

### 2.1. Isolation of Bacteria

A total of 421 GNB isolates, which included multiple distinct isolates from the same samples, were obtained from 401 milk samples. To ensure a comprehensive study, all different isolates were included, resulting in an increased isolate count of 421 (Table 1).

### 2.2. Antibiotic Susceptibility Testing (AST)

Two-hundred-ninety-five antibiotic resistant isolates were found among 421 total isolates screened by the disc diffusion test (DDT). Significantly (*p* = 0.003) more isolates in milk from Assam were found to be antibiotic resistant (77%, CI (70–82)) versus (63%, CI (56–70)) compared to Haryana. There was no difference in the number of resistant isolates in milk from vendors or farmers (Table 2). Only 1% (CI (0.2–4)) of the isolates in the milk from Haryana and none of the isolates from Assam were resistant to all six antibiotics (Table 3).

### 2.3. Polymerase Chain Reaction (PCR) Detection of Resistance Genes

All the isolated bacteria (n = 421) were subjected to multiplex PCR, to screen for selected ESBL, MBL, and AmpC producing genes using 17 PCR primer pairs. The PCR identified 43 isolates as producers of β-lactamases. Among these, 9 isolates were found to carry genes associated with ESBL, 11 isolates carried genes associated with MBL, and 28 isolates carried genes associated with AmpC. Notably, certain isolates exhibited a combination of resistance genes, involving ESBL, MBL, or AmpC. Overall, the prevalence of β-lactamases was found to be 10% in total, with 13% prevalence in Haryana, and 7% prevalence in Assam. However, a similar prevalence (11 vs. 10%) was observed among the isolates from milk from farmers and from vendors.

Most of the resistant isolates from milk of Haryana harbored an AmpC associated genes (10%, CI (6–15)), next most common was ESBL (3%, CI (1–6)) and then MBL (2%, CI (0.7–5)) associated genes. Similarly, isolates from milk from Assam more commonly had AmpC (3%, CI (1–6)), followed by MBL (3%, CI (1–6)) and ESBL (1%, CI (0.2–4)) associated genes. Within the group of 43 PCR-positive isolates, there were 5 isolates displaying combinations of resistance genes. Specifically, there was one isolate each with the combination of ESBL + AmpC and ESBL + MBL, observed in isolates sourced from milk in Haryana. Additionally, three isolates exhibited the AmpC + MBL combination (n = 3) and originated from Assam (Table 4).

The observed β-lactamase genes in milk isolates were bla_CMY_ (n = 13), bla_MOX_ (n = 10), bla_FOX_ (n = 6), bla_EBC_ (n = 2), and bla_DHA_ (n = 1), associated with AmpC producers. Additionally, bla_VIM_ (n = 5), bla_IMP_, and bla_SPM_ (n = 4), bla_SIM_ (n = 3), and bla_GIM_ (n = 1) were detected, which are found in MBL producers. Furthermore, bla_CTX-M1_ (n = 7), bla_SHV_, and bla_TEM_ (n = 1) were detected, which are associated with ESBL producers (Supplementary Materials).

The 43 isolates that were confirmed as β-lactamases (ESBL, MBL and AmpC) by the PCR genotyping method were further identified at the genus level as *Shigella* spp. (n = 12), *Klebsiella* spp. (n = 6), and *Escherichia coli* (*E. coli*) (n = 2) with 23 “other Enterobacteriaceae” that were not identified further (Table 5).

The genus *Shigella* spp. were found carrying seven AmpC, six MBL and one ESBL associated gene. Similarly, the *Klebsiella* spp. carried five AmpC and two ESBL associated genes, whereas the *E. coli* was found to carry only one each of MBL and ESBL associated genes. Among the other Enterobacteriaceae, the majority of the isolates were found to carry genes associated with AmpC (n = 17) followed by ESBL (n = 5) and MBL (n = 4) genes (Table 5).

It is noteworthy that one isolate from pasteurized milk was positive for bla_FOX_ and bla_CMY,_ genes associated with AmpC, while the other genes were found only in isolates from raw milk. Since the isolated bacteria from pasteurized milk carrying the AmpC associated genes was not identified, it was classified as “other Enterobacteriaceae”.

### 2.4. Comparison between Results

The results of the Epsilometer test (E-test) and the DDT were compared to the genotypically confirmed isolates carrying the β-lactamases genes to check the sensitivity and consistency of the phenotypic tests (DDT and E-test) in detecting β-lactamases. Among the genotypically confirmed β-lactamase producing isolates, the DDT showed 68% (CI (48–84)) and 57% (CI (37–76)) resistance for cefoxitin and cefotetan, whereas the E-test showed 71% (CI (51–87)) and 46% (CI (28–66)) for the respective antibiotics. In detecting ESBL, DDT showed 67% (CI (30–92)) and 44% (CI (14–79)) resistance to cefotaxime and ceftazidime, whereas the E-test showed 44% (CI (14–79)) and 33% (CI (7–70)), respectively. For detecting MBL, DDT showed 9% (CI (0.2–41)) and 0% resistance to imipenem and meropenem, whereas the E-test showed 9% (CI (0.2–41)) for both the antibiotics (Table 6).

The DDT was found to detect more β-lactam resistance than the E-test among the isolates with resistance genes. For the genotypically confirmed isolates with MBL genes, the DDT showed 73% (CI (39–94)) of isolates to be positive for MBL, whereas the E-test showed only 36% (CI (11–69)) of isolates as MBL. For the genotypically confirmed isolates with AmpC genes there were slight differences between the DDT and E-test (79%, CI (59–92) vs. 75%, CI (55–89)), respectively. However, the E-test was found to be slightly more sensitive than the DDT in detecting phenotypic resistance in isolates with confirmed ESBL genes (78%, CI (40–97) vs. 67%, CI (30–93)) (Table 7). The AST results for the quality control strain *Escherichia coli* ATCC 25922, were between the acceptable range as per CLSI 2015 [45] (Supplementary Materials).

## 3. Discussion

In the current study we found 43 GNB harboring β-lactamase genes, and the overall prevalence of β-lactamases by PCR genotyping in milk was 10% (CI (7–13)). However, this was lower than the previous reports (22–30%) in milk [46,47], probably because the milk samples in our studies were obtained from points of sale meant for consumption.

The prevalence of genotypic β-lactamases was 13% (CI (9–19)) in Haryana (a developed dairy sector) and 7% (CI (4–11)) in Assam (mainly informal dairy sector). These findings could indicate that intensive farms have a higher prevalence of antibiotic resistance genes, which has also been reported by others [48,49] and was similarly found in our previous study on Gram-positive bacteria in milk [50]. Even though we observed a higher prevalence of β-lactamase genes in milk isolates from Haryana compared to isolates from Assam, we discovered that milk isolates from Assam (77%, CI (70–82)) exhibited a higher phenotypic β-lactam resistance compared to isolates from Haryana (63%, CI (56–70)), and we therefore cannot conclude on the difference between the states. This disagreement further indicates that factors other than the presence of these β-lactamase genes may contribute to the observed phenotypic resistance.

Among the genotypically confirmed β-lactamase producing isolates, the most dominant bacteria identified, besides “other Enterobacteriaceae” (53%, CI (38–69)) was *Shigella* spp. (28%, CI (15–44)), next was *Klebsiella* spp. (14%, CI (5–28)), then *E. coli* (5%, CI (0.5–16)) (Table 7). The isolated *Shigella* spp. outnumbered other identified species and exhibited a varied antimicrobial resistance pattern that included resistant genes associated with AmpC, MBL and ESBL, including resistance in combination. The increase in disease associated with *Shigella* spp. is contributing to a great burden of hospitalizations due to food poisoning, making it of significant concern to public health in India and beyond [51,52,53,54].

However, global surveillance investigations have revealed high frequencies (exceeding 50%) of *E. coli* and *Klebsiella* spp. producing β- lactamases in Asia, Africa, and Latin America [15]. The presence of β-lactamases among *Shigella* spp., *Klebsiella* spp., and *E. coli*, is a significant concern as it has been linked to numerous outbreaks of foodborne illnesses in humans. This finding highlights a potential foodborne public health hazard [55,56,57,58,59,60]. Understanding the prevalence and resistance profile of Enterobacteriaceae in dairy settings is crucial for implementing effective control measures and safeguarding public health.

The combination of antibiotic resistance genes (ARG’s) associated with AmpC + MBL was detected in isolates from both Haryana and Assam. However, combined ARG’s associated with ESBL + AmpC and ESBL + MBL were detected in isolates from Haryana only (Table 5). Even though the co-occurrence of β-lactamases enzymes, especially AmpC and ESBLs, is a common phenomenon [61], the presence of combined ARGs from AmpC + MBL, as observed in our study with *Shigella* spp., is rare and most likely the result of plasmid circulation within strains from different environments [62]. However, we did not conduct a genomic study to determine the specific genomic location of the resistant genes to establish the presence of plasmid circulation in isolates.

Among the β-lactamase genes found in Enterobacteriaceae, bla_CTX-M1_, bla_SHV_, bla_TEM_, bla_CTX-M3_ were previously identified in Indian cow milk. Additionally, a few also reported the presence of bla_NDM,_ bla_CTX-M9_ and bla_CTX-M15_ genes in cow milk in India [63,64,65,66,67]. These genes grant resistance to bacterial isolates against β-lactam antibiotics such as penicillin and cephalosporin, commonly observed in clinical settings and often associated with human infections [68,69].

The AmpC-associated genes that have been identified in this study, namely bla_CMY_, bla_MOX_, bla_FOX_, bla_EBC_, and bla_DHA_, have been previously documented in both humans and animals in certain countries, including India [57,70,71,72,73,74,75,76].

In contrast, the occurrence of bla_VIM_, bla_IMP_, bla_SPM_, bla_SIM_, and bla_GIM_ genes associated with MBL is a relatively rare phenomenon, and these genes have not been previously reported in dairy animals within the country. The observation of these genes in our study raises intriguing questions about the mechanisms by which these genes or pathogens enter the dairy value chain, especially given the limited reports of MBL resistance in humans. One study reported the emergence of plasmid-mediated resistance, including ESBL, AmpC, and MBL, in wild animals, which were regarded as indicators of environmental antibiotic resistance contamination [77].

It is worth noting that there have been no prior comprehensive studies conducted in India and elsewhere that focused on investigating the existence of β-lactamase genes in milk intended for human consumption, however, a study carried out in Indonesia also undertook a similar investigation involving bulk tank milk [78]. The β-lactamase genes are recognized for their association with antibiotic resistance, rendering bacterial strains carrying them less susceptible to important antibiotics. The clinical significance of these genes lies in their potential to undermine the effectiveness of antibiotic treatments, making infections caused by bacteria harboring these genes more difficult to manage [79,80,81]. This can lead to a higher likelihood of treatment failure causing increased morbidity, longer hospital stays, and higher case fatality.

The presence of MBL/AmpC/ESBL-associated genes in milk isolates underscores the need for additional research to uncover the routes by which these genes enter the dairy value chain. Exploring their spread mechanisms within and beyond this chain is a complex process and requires further investigation. Although certain antibiotic-resistant bacteria might naturally exist in animals and their environment, misuse of antibiotics in agriculture can enhance the selection and endurance of resistant strains [77]. The possibility of these genes transferring from livestock to humans through consumption of contaminated dairy products underscores the importance of strict hygiene protocols and responsible antibiotic management in both animal husbandry and food production.

We found a low concordance between the genotyping and phenotyping methods. Among the PCR confirmed β-lactamases (n = 43) from Assam and Haryana, 63% (CI (47–77)) isolates showed resistance by both the DDT and the E-test. Notably, eight isolates found negative by both DDT and the E-test were positive by PCR. Some investigations also highlight inconsistent sensitivity of different phenotypic techniques [82]. Our results indicate that the DDT is slightly more sensitive than the E-test and supports the idea that the E-test alone is not a reliable method for the detection of β-lactamases [83]. Thus, the present study demonstrated variations in concordance between the two phenotypic tests and the confirmed β-lactamase isolates identified through the PCR genotyping method.

In our analysis, the PCR genotyping method detected a 10% prevalence of ARGs, which is significantly lower when compared to the prevalence observed using the DDT (70%) as the phenotypic method. These substantial differences in detecting β-lactamases suggest that resistance genes may not always be actively expressed, and there could be other mechanisms of resistance involved (not explored in this study). Thus, a comprehensive approach combining both phenotypic and genotypic testing offers a more accurate understanding of antibiotic resistance, helping inform treatment strategies and control measures.

We found that while most of the AmpC-producing isolates were resistant to second-generation cephalosporins, some were also resistant to third generation cephalosporins and carbapenems, as indicated by both the DDT and E-test results. Similarly, although ESBL-producing isolates were primarily resistant to third generation cephalosporins, some were also found to be resistant to carbapenems. Additionally, among the MBL-producing isolates, most isolates exhibited resistance to carbapenems, and a significant number of them also displayed resistance to third generation cephalosporins.

In a populated nation such as India, resistance to third-generation cephalosporins presents a significant challenge to control the rapid rise in infection caused by resistant bacteria [84]. In developed nations such as in Europe, carbapenems are banned from being used in food producing animals while third and fourth generation cephalosporins are approved only in cattle and pigs [85]; but making similar restrictions in LMIC is a challenge [86,87,88] owing to poor regulation and unrestricted sale of antibiotics.

It is generally known that pasteurization kills most bacteria [89], and the milk in India goes through ultra-high temperature (UHT) treatment for pasteurization. However, the discovery of one antibiotic-resistant isolate (“other Enterobacteriaceae”) from pasteurized milk harboring the AmpC associated gene is alarming. To our knowledge, no other study from India has reported β-lactamases in GNB isolated from pasteurized milk. This finding aligns with our previous report, which identified methicillin-resistant Gram-positive bacteria in pasteurized milk [50]. It could also be due to post-pasteurization contamination, potentially exacerbated by inadequate quality packaging and cold storage that encourages bacterial growth. However, one study reported that some bacteria, especially those with antibiotic resistance genes, may not always be eliminated unless an efficient pasteurization technique is used [90]. In India, milk is used in a variety of products such as cheese, yoghurt, sweets etc., and these are consumed widely. The antimicrobial resistance in milk can have an impact on the entire dairy food manufacturing chain [90]. The fact that several microorganisms can contaminate milk, there is indiscriminate use of antimicrobials in milk production, together with a lack of hygiene during milk processing and storage, are of great concern [91,92].

Additionally, the rising global population and economic prosperity are both contributing to a rising demand for animal-sourced food [93]. Therefore, it is not surprising that livestock industries use more antibiotics than are used for humans [94]. Moreover, during the past ten years, use of antibiotics in animal agriculture has increased by 70% driven mainly by an increase in global demand for dairy, fish, eggs and meat [10,95]. Hence, more emphasis should be given to sensitizing the value chain actors about antibiotic usage and associated risks in food animals [7,96,97].

However, the study was limited by the relatively small sample size utilized, which may affect the generalizability of our findings. It is crucial to note that the analysis was confined to pasteurized milk from a single Indian state, Haryana. This limited scope could introduce biases for the broader applicability of our results. Furthermore, while the broader category of “other Enterobacteriaceae” was identified as β-lactam producers we did not identify them further. If future studies could include a more extensive sample size for the analysis of pasteurized milk and identify all the β-lactamase producing Enterobacteriaceae, it would yield a more accurate portrayal of the magnitude of the resistant bacteria, as well as shed light on potential entry points for contamination post-pasteurization. Exploring viable solutions necessitates an enlarged dataset to yield meaningful insights.

The targeted β-lactamase gene variants we selected may not be representative of all possible variants, potentially leading to an underestimation of the prevalence of ARGs, as it may not encompass the entire spectrum of possible variants. In forthcoming studies, we recommend adopting a broader approach such as sequencing of amplified fragments to avoid potential occurrence of false positive genes to ensure a more comprehensive representation of ARGs.

In shaping the trajectory of future investigations, we propose a multi-faceted approach. Firstly, expanding the sample size to include representative states heavily involved in cattle milk production and distribution would provide a more comprehensive view of the issue. Moreover, performing a thorough risk assessment is crucial for accurately assessing the potential repercussions on public health, especially concerning antibiotic resistance within food and the associated risks linked to food consumption. An intriguing avenue for further exploration lies in the development of a model for human risk assessment associated with AMR in animal-derived food products. Such a model could offer insights into the potential ramifications of AMR on human health, guiding regulatory decisions and facilitating effective risk management strategies.

The significance of this study extends beyond India’s borders. With the growing influence of globalization and trade, the widespread use of antibiotics and the rise of AMR poses a global public health concern. Thus, action is imperative to address this challenge on a worldwide scale.

## 4. Materials and Methods

### 4.1. Ethical Statement

Ethical approval for the study was granted by the Institutional Research Ethics Committee (IREC) of the International Livestock Research Institute (ILRI) on 21 September 2015 (No. ILRI-IREC2015-12) and 27 February 2017 (No. ILRI-IREC2017-05) and approved by the collaborating institutes from the Indian Council of Agricultural Research (ICAR).

### 4.2. Sample Collection

**Figure 1:**
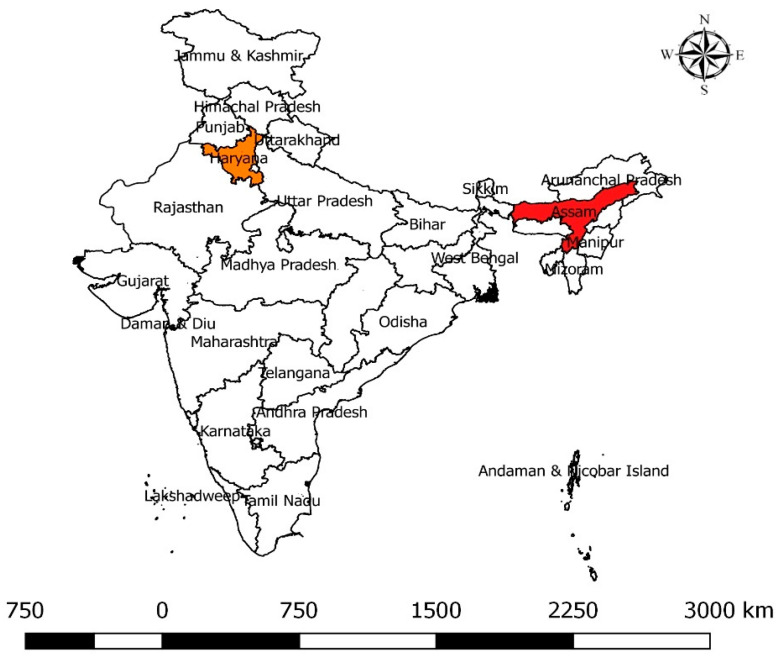
Sampling states under the spotlight: Assam marked in red and Haryana in orange on the Map of India.

A cross-sectional study was conducted in two Indian states—Assam and Haryana (Figure 1). The two states were chosen based on the differences in geography, infrastructure, and dairy development. Haryana is located at the center of north India, whereas Assam is situated in the north-eastern part. Assam has an unorganized (informal) dairy system with many local-breed dairy animals while Haryana has a more developed dairy sector with many high yielding cattle and buffalo [98].

The sample size calculation was performed using a one-sample binomial calculation, assuming a 95% level of confidence, 5% level of precision, and assuming that 15% of the samples contained resistant bacteria. This yielded a result of about 200 samples per state [99]; to account for a small design effect, we aimed for 240 samples. The study finally involved the collection of 401 milk samples, with 222 samples obtained from Haryana and 179 samples from Assam. The samples were sourced from two distinct groups, dairy farmers (n = 242) and vendors (n = 137). For the dairy farmer samples, milk samples were collected from specific districts in Haryana and Assam. In Haryana, the samples were collected from the districts of Karnal, Bhiwani, and Kaithal. In Assam, the samples were collected from the districts of Golaghat, Baska and Kamrup during December 2016 to February 2017. Regarding the vendor samples, two categories were distinguished, raw milk and pasteurized milk. For Haryana, both raw and pasteurized milk samples were collected from the districts of Karnal, Bhiwani, and Kaithal. In Assam, only raw milk samples were collected from the same districts, with the addition of the Kokrajhar district. The collection period for the vendor samples in both states was from September to November 2017.

The sampling process involved a multi-level random sampling method that encompassed villages, dairy farms, milk traders, and vendors within the selected districts as had been previously described in detail in previous publications. Among the vendor samples, 22 were pasteurized milk samples obtained from milk retail outlets or grocery stalls, while the remaining vendor samples were raw milk collected from milk vendors (Table 1). The study collected milk at the point of consumption or sale for consumption, including both cow and buffalo milk.

The study collected 10 mL milk samples aseptically in sterile 50 mL Falcon tubes (Tarson, Kolkata, India). These samples were obtained from two primary sources, bulk milk cans at dairy farms for consumption and milk cans held by vendors for sale. Additionally, packaged pasteurized milk was purchased in pouches from vendors to represent the associated public health risk with milk consumption. The samples were then transported to the laboratory while maintaining a cold chain and kept at 4 °C until processing. The duration between sample collection and the commencement of analysis did not exceed 48 h.

### 4.3. Isolation of Bacteria

**Figure 2:**
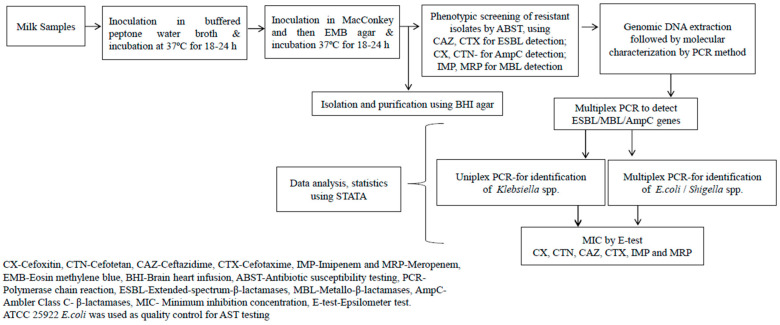
Flow chart representing the isolation of Gram-negative bacteria, phenotypic and genotyping screening of β-lactamases (ESBL, AmpC, MBL) and determination of MIC by the Epsilometer test (E-test).

The samples were processed by inoculating in buffered peptone water (Hi-media, Maharashtra, India) and incubated at 37 °C for 18–24 h to grow presumptive GNB. The culture broth was inoculated on MacConkey agar plates (Hi-media, Maharashtra, India) and incubated at 37 °C for 18–24 h to grow presumptive *E. coli*, *Shigella* spp. and *Klebsiella* spp. Suspected *E. coli* were further inoculated in Eosin Methylene Blue (EMB) agar (Hi-media, Maharashtra, India) for differential detection. Brain heart infusion agar (Hi-media, Maharashtra, India) was used for subculturing and purification. The colonies were initially identified based on colony morphology, Gram staining and biochemical tests. If morphologically distinct colonies were present from a single sample, they were considered as separate isolates for further analysis, meaning that there were more isolates than original samples according to the schematic represented in Figure 2.

The purified bacterial isolates were subjected to antibiotic susceptibility testing (AST) by a DDT using six antibiotics (Table 8).

### 4.4. Antibiotic Susceptibility Testing (AST)

All the purified isolates were subjected to AST for phenotypic detection of β-lactam resistance using the Kirby–Bauer DDT [100] method following the guidelines of the Clinical and Laboratory Standards Institute [45]. Prior to AST, a bacterial cell suspension in normal saline solution (0.85%) was made and the turbidity was set to 0.5 McFarland units. A sterile cotton swab was dipped into the broth culture tube and rotated several times to get an adequate amount of culture and uniformly spread on the surface of the Mueller–Hinton Agar (MHA) (Hi-media, Maharashtra, India) plates. The antibiotic discs (Hi-media, Maharashtra, India) were placed on the cultured MHA plates. Within 15 min of placing the antibiotic discs on the cultured plates, the plates were incubated at 37 °C for 18–24 h. The plates were then examined for confluent growth and circular zones of inhibition around the antibiotic discs were measured according to the manufacturer’s instruction. As outlined in Table 8, for the purpose of this study, the isolates were classified as ESBL if resistant to cefotaxime and ceftazidime. Similarly, the isolates were classified as AmpC if resistant to cefoxitin and cefotetan; and as MBL if resistant to imipenem and meropenem. Isolates that were found resistant to any single tested antibiotic were designated as “resistant isolates”. *Escherichia coli* ATCC 25922 was used as a quality control.

### 4.5. Molecular Detection of Resistance Genes by PCR

Genomic DNA of the isolates was extracted using a DNA extraction kit (Qiagen, Germantown, USA). PCR was performed to detect the β-lactamase producing genes. The target genes bla_TEM_, bla_SHV_, bla_CTX-M1_, bla_CTX-M2_, bla_CTX-M3_, bla_CTX-M4_ were used for ESBL detection; bla_IMP_, bla_VIM_, bla_GIM_, bla_SIM_, bla_SPM_ for MBL detection and bla_FOX_, bla_MOX_, bla_EBC_, bla_ACC_, bla_DHA_, bla_CMY_ for AmpC detection. The amplicons were regarded as positive for the specific β-lactam resistance gene if any single bands appeared post-gel electrophoresis and referred to here as being genotypically resistant to differentiate from the phenotypic results.

When isolates were found positive for any of the β-lactamase genes associated with ESBL, MBL or AmpC by PCR, the isolates were further identified for *Shigella* spp., *Klebsiella* spp., and *E. coli* using PCR (Table 9) to confirm the preliminary identification that was performed based on colony morphology, Gram staining and biochemical tests. The isolates were categorized as “other Enterobacteriaceae”, if they could not be confirmed as *Shigella* spp., and *Klebsiella* spp., or *E. coli*. To verify the results, the PCR was repeated twice for all samples and a positive control was always utilized for PCR confirmation. *Shigella* spp., and *Klebsiella* spp., and *E. coli* were selected as they are important pathogens in cows and have been found in milk in an earlier report on dairy farms of Kenya [101].

### 4.6. Epsilometer Test

All the PCR confirmed β-lactamase isolates were subjected to an E-test to determine the MIC required to inhibit/kill the bacteria [45]. To perform an E-test, a bacterial cell suspension was made in normal saline solution (0.85%) and the turbidity was set equivalent to 0.5 McFarland units [100]. A sterile cotton swab was dipped into the broth culture tube and rotated several times to get an adequate amount of culture; it was then uniformly applied on the surface of the MHA (Hi-media, Maharashtra, India) plate. The antibiotic (Hi-media, Maharashtra, India) strips were placed on the MHA agar plate, using sterile forceps, by gently pressing the antibiotic strips to ensure their complete contact with the surface of the agar plate. The inoculation was performed within 10–15 min of the inoculum being prepared in normal saline. The plates were then incubated at 37 °C for 16–20 h, and then examined for the MIC value from the scale in terms of µg/mL where the ellipse edge intersects the strip. *Escherichia coli* ATCC 25922 was used as quality control and results were in the acceptable range in all runs.

## 5. Conclusions

This study provides compelling evidence of antibiotic-resistant bacterial contamination in milk intended for human consumption. The presence of β-lactamases, including ESBL, AmpC, and MBL, among the isolated *E. coli, Shigella* spp., *Klebsiella* spp., and other Enterobacteriaceae in dairy milk at the point of consumption poses a significant public health risk. The organized dairy sector seems more likely to exhibit AMR genes, which may be attributed to the consequent treatment pressure on animals due to rising demand for milk and milk products. The observed disparity between genotypic and phenotypic methods highlights the importance of comprehensive approaches that integrate both techniques for accurate assessment of antibiotic resistance. The emergence of β-lactam resistance presents a formidable challenge globally, particularly in India, highlighting the urgent need for joint action involving all the value chain actors.

These findings underscore the importance of responsible antibiotic use, establishment of stringent regulations, strict monitoring, implementation of pasteurization, protection of the milk meant for consumption and implementation of proper hygiene practices to avoid contamination post-pasteurization that would greatly impact food safety and public health to mitigate the emergence and spread of antibiotic resistance in the dairy sector. Further, risk assessment is essential to establish the safety of public health and must be performed in future studies.

## Figures and Tables

**Table 1:** Numbers of bacterial isolates and milk samples collected in Assam and Haryana, India.

Milk Source	Sample Type	Assam	Haryana	Total
Milk from dairy farmer	Raw milk	116	126	242
	Sample positive *	113	112	225
	Isolate	148	115	263
Milk from dairy vendor	Raw milk	63	74	137
	Sample positive *	63	73	136
	Isolate	63	73	136
	Pasteurized milk	0	22	22
	Sample positive *	0	22	22
	Isolates	0	22	22
	Total sample	179	222	401
	Total positive *	176	207	383
	Total isolate	211	210	421

* Positive sample represents at least one isolated bacterial isolate in the sample.

**Table 2:** Gram-negative bacterial isolates resistant to β-lactam antibiotics by the disc diffusion test.

	Total Isolates (n = 421)	Isolates from Haryana (n = 210)	Isolates from Assam (n = 211)	*p*-Value	Isolates from Farmers (n = 263)	Isolates from Vendors (n = 158)	*p*-Value
	n (%)	n (%)	n (%)		n (%)	n (%)	
Resistant isolates	295 (70.07)	133 (63.33)	162 (76.78)	0.003	183 (69.58)	112 (70.89)	0.826
Non-resistant isolates	126 (29.93)	77 (36.67)	49 (23.22)		80 (30.42)	46 (29.11)	

**Table 3:** Gram-negative bacterial isolates resistant and susceptible to a number of β-lactam antibiotics by the disc diffusion test.

Milk Sources	Isolates						
	Resistant to 6 Antibiotics	Resistant to 5 Antibiotics	Resistant to 4 Antibiotics	Resistant to 3 Antibiotics	Resistant to 2 Antibiotics	Resistant to 1 Antibiotics	Sensitive Isolates
	n (%)	n (%)	n (%)	n (%)	n (%)	n (%)	n (%)
Assam (n = 211)	0	4 (1.90)	20 (9.48)	31 (14.69)	49 (23.22)	58 (27.49)	49 (23.22)
Haryana (n = 210)	3 (1.43)	2 (0.95)	15 (7.14)	29 (13.81)	38 (18.10)	46 (21.90)	77 (36.67)
Farmer (n = 263)	2 (0.76)	3 (1.14)	18 (6.84)	33 (12.55)	57 (21.67)	70 (26.62)	80 (30.42)
Vendor (n = 158)	1 (0.63)	3 (1.90)	17 (10.76)	27 (17.09)	30 (18.99)	34 (21.52)	46 (29.11)
Total (n = 421)	3 (0.71)	6 (1.43)	35 (8.31)	60 (14.25)	87 (20.67)	104 (24.70)	126 (29.93)

**Table 4:** Isolates positive for β-lactamase genes by the PCR genotyping method.

Milk Source	β-Lactamases Positive	* ESBL Genes Positive	* MBL Genes Positive	* AmpC Genes Positive	AMR Genes in Combination (n = 421)		
					AmpC + MBL	ESBL + AmpC	ESBL + MBL
	n (%)	n (%)	n (%)	n (%)	n (%)	n (%)	n (%)
Assam (n = 211)	15 (7.11)	3 (1.42)	6 (2.84)	7 (3.31)	1 (0.23)	0	0
Haryana (n = 210)	28 (13.33)	6 (2.86)	5 (2.38)	21 (10.00)	2 (0.47)	1 (0.23)	1 (0.23)
Total (n = 421)	43 (10.21)	9 (2.13)	11 (2.61)	28 (6.65)	3 (0.71)	1 (0.23)	1 (0.23)
Farmer (n = 263)	28 (10.65)	6 (2.28)	10 (3.80)	16 (6.08)	3 (0.71)	1 (0.23)	1 (0.23)
Vendor (n = 158)	15 (10.13)	3 (1.89)	1 (0.63)	12 (7.59)	0	0	0
Total (n = 421)	43 (10.21)	9 (2.13)	11 (2.61)	28 (6.65)	3 (0.71)	1 (0.23)	1 (0.23)

* Including AMR genes in combination.

**Table 5:** Identification of bacterial isolates confirmed to harbor β-lactamase genes by PCR.

Milk Source	*E. coli*	*Shigella* spp.	*Klebsiella* spp.	Other Enterobacteriaceae	Total Isolates
	n (%)	n (%)	n (%)	n (%)	n
Assam (n = 15)	0	10 (66.67)	0	5 (33.33)	15
Haryana (n = 28)	2 (7.14)	2 (7.14)	6 (21.43)	** 18 (64.29)	28
Total (n = 43)	2 (4.65)	12 (27.90)	6 (13.95)	23 (53.48)	43
Milk source	*E. coli*	*Shigella* spp.	*Klebsiella* spp.	Other Enterobacteriaceae	Total isolates
	n (%)	n (%)	n (%)	n (%)	n
Farmer’s (n = 28)	2 (7.14)	12 (42.86)	4 (14.29)	10 (35.71)	28
Vendor’s (n = 15)	0	0	2 (13.33)	** 13 (86.67)	15
Total (n = 43)	2 (4.65)	12 (27.90)	6 (13.95)	23 (53.48)	43
β-Lactamases	*E. coli*	*Shigella* spp.	*Klebsiella* spp.	Other Enterobacteriaceae	* Total Genes Identified
	n (%)	n (%)	n (%)	n (%)	n
AmpC genes * (n = 28)	0	7 (25.00)	5 (17.85)	17 ** (60.71)	28
MBL genes * (n = 11)	1 (9.09)	6 (54.54)	0	4 (36.36)	11
ESBL genes * (n = 9)	1 (11.11)	1 (11.11)	2 (18.18)	5 (55.55)	9

* Including AMR genes in combination ** Including one pasteurized milk sample.

**Table 6:** Genotypically confirmed β-lactamase isolates and their phenotypic resistance by the disc diffusion test (DDT) and Epsilometer test (E-test).

Antibiotic Classes	Phenotypic Resistance by DDT	ESBL Positive Genes # (n = 9)	MBL Positive Genes * (n = 11)	AmpC Positive Genes $ (n = 28)	Phenotypic Resistance by E-Test	ESBL Positive Genes # (n = 9)	MBL Positive Genes * (n = 11)	AmpC Positive Genes $ (n = 28)
		n (%)	n (%)	n (%)		n (%)	n (%)	n (%)
2nd generation cephalosporin (AmpC Resistance $)	Resistant to cefoxitin	5 (55.56)	6 (54.54)	19 (67.86)	Resistant to cefoxitin	3 (33.33)	3 (27.27)	20 (71.43)
	Resistant to cefotetan	4 (44.44)	4 (36.36)	16 (57.14)	Resistant to cefotetan	3 (33.33)	3 (27.27)	13 (46.43)
3rd generation cephalosporin (ESBL Resistance #)	Resistant to cefotaxime	6 (66.67)	6 (54.55)	6 (21.43)	Resistant to cefotaxime	4 (44.44)	3 (27.27)	12 (42.86)
	Resistant to ceftazidime	4 (44.44)	4 (36.36)	9 (32.14)	Resistant to ceftazidime	3 (33.33)	0	12 (42.86)
Carbapenem-β-lactam (MBL Resistance *)	Resistant to imipenem	1 (11.11)	1 (9.09)	1 (3.57)	Resistant to imipenem	1 (11.11)	1 (9.09)	1 (3.57)
	Resistant to meropenem	1 (11.11)	0	0	Resistant to meropenem	1 (11.11)	1 (9.09)	7 (25)

$—AmpC-β-lactamase, #—Extended spectrum-β-lactamase, *—Metallo-β-lactamase.

**Table 7:** Proportion of β-lactamases detected by the genotypic method and β-lactam resistance by phenotypic methods; disc diffusion test (DDT) and Epsilometer test (E-test).

Milk Source	Isolates		
	PCR Positive #	Resistant by DDT	Resistant by E-Test
	n (%)	n (%)	n (%)
Assam	15/43 (34.88)	13/15 (86.67)	8/15 (53.33)
Haryana	28/43 (65.11)	20/28 (71.43)	21/28 (75.00)
Total	43/43 (100.00)	33/43 (76.74)	29/43 (67.44)
β-lactamases		Isolates	
	AMR genes *	Resistant by DDT	Resistant by E-test
	n	n (%)	n (%)
ESBL genes (n = 9)	9	6 (66.67)	7 (77.78)
MBL genes (n = 11)	11	8 (72.73)	4 (36.36)
AmpC genes (n = 28)	28	22 (78.57)	21 (75.00)
Total	43	36 (83.72)	32 (74.41)

* Including AMR genes in combination (n = 5), # individual isolates.

**Table 8:** β-lactam antibiotics with classes and concentrations used in a disc diffusion test (DDT) and in the E-test to determine minimum inhibitory concentration.

Classes	β-Lactam Antibiotics		Concentration of Antibiotic Disc (in µg)	Breakpoints for Resistance by DDT (mm)	Concentration of Antibiotic Strips (µg/mL)	Breakpoints for Resistance by E-Test (µg/mL)
3rd generation cephalosporins	For ESBL detection	Cefotaxime Ceftazidime	30 30	≤22 ≤17	0.016–256 0.016–256	>4 >16
2nd generation cephalosporins	For AmpC detection	Cefoxitin Cefotetan	30 30	≤14 ≤12	0.016–256 0.016–256	>32 >64
Carbapenem-β-lactams	For MBL detection	Imipenem Meropenem	30 30	≤19 ≤19	0.002–32 0.002–32	≥4 >4

**Table 9:** PCR primer details for identifying β-lactamases (ESBL, MBL and AmpC) genes and identification of bacterial species.

Gene	Forward (5′-3′)	Reverse (5′-3′)	Length (* bp)	* AT	* Ref.
ESBL genes				60 °C	[102,103,104]
blaTEM	ATGAGTATTCAACATTTTCG	TTACCAATGCTTAATCAGTG	861		
blaSHV	ATGCGTTATATTCGCCTGTG	TTAGCGTTGCCAGTGCTCGA	860		
blaCTXM1	GACGATGTCACTGGCTGAGC	AGCCGCCGACGCTAATACA	499		
blaCTXM2	GCGACCTGGTTAACTACAATCC	CGGTAGTATTGCCCTTAAGCC	351		
blaCTXM3	CGCTTTGCCATGTGCAGCACC	GCTCAGTACGATCGAGCC	307		
blaCTXM4	GCTGGAGAAAAGCAGCGGAG	GTAAGCTGACGCAACGTCTG	474		
MBL genes				53 °C	[105]
blaIMP	GAATAG(A/G)(A/G)TGGCTTAA(C/T)TCTC	CCAAAC(C/T ACTA(G/C)GTTATC	188		
blaVIM	GTTTGGTCGCATATCGCAAC	AATGCGCAGCACCAGGATAG	382		
blaGIM	TCAATTAGCTCTTGGGCTGAC	CGGAACGACCATTTGAATGG	72		
blaSIM	GTACAAGGGATTCGGCATCG	TGGCCTGTTCCCATGTGAG	569		
blaSPM	CTAAATCGAGAGCCCTGCTTG	CCTTTTCCGCGACCTTGATC	798		
AmpC genes				64 °C	[106]
blaFOX	AACATGGGGTATCAGGGAGATG	CAAAGCGCGTAACCGGATTGG	190		
blaMOX	GCTGCTCAAGGAGCACAGGAT	CACATTGACATAGGTGTGGTGC	520		
blaEBC	TCGGTAAAGCCGATGTTGCGG	CTTCCACTGCGGCTGCCAGTT	302		
blaACC	AACAGCCTCAGCAGCCGGTTA	TTCGCCGCAATCATCCCTAGC	346		
blaDHA	AACTTTCACAGGTGTGCTGGGT	CCGTACGCATACTGGCTTTGC	405		
blaCMY	TGGCCAGAACTGACAGGCAAA	R TTTCTCCTGAACGTGGCTGGC	462		
*E. coli*				60 °C	[107,108]
lacY	CTACCGGTGAACAGGGTAGC	GTCGCTGAAAAACGCACTTC	289		
lacZ	ATGAAAGCTGGCTACAGGAAGG	CTCCACACAGTTTCGGGTTTTC	517		
cyd	CCGTATCATGGTGGCGTGTGG	GCCGGCTGAGTAGTCGTGGAAG	398		
uidA	CGCCGATGCAGATATTCG	GCTGTGACGCACAGTTCATAG	603		
phoA	GGTAACGTTTCACCGCAGAGTTG	CAGGGTTGGTACACTGTCATTACG	468		
*Shigella* spp.					
phoA	GGTAACGTTTCACCGCAGAGTTG	CAGGGTTGGTACACTGTCATTACG	468		
*Klebsiella* spp.				62 °C	[109]
gyrA	CGCGTACTATACGCCATGAACGTA	ACCGTTGATCACTTCGGTCAGG	441		

* AT—Annealing Temperature, bp—base pairs, Ref.—References.

## Data Availability

Data is provided in Supplementary Material.

## Supplementary Materials

The following supporting information can be downloaded at: https://www.mdpi.com/2079-6382/12/9/1449/s1, Table S1: Details of milk samples, results of antibiotic-resistance profile for the isolated Gram-negative bacteria, their molecular characterization and the AST result for the QC *Escherichia coli* ATCC 25922.
